# Targeted Cauterization of Bleeding Cervical Polyps During Pregnancy: A Case Series

**DOI:** 10.1002/ccr3.73245

**Published:** 2026-07-27

**Authors:** Satoshi Yoneda, Noriko Yoneda, Tatsuhiro Tsuda, Masami Ito, Sayaka Tsuda, Kyoko Takemura

**Affiliations:** ^1^ Department of Obstetrics and Gynecology University of Toyama Toyama Japan

**Keywords:** bleeding cervical polyp, cauterization, decidual cervical polyp, minimally invasive approach, pregnancy

## Abstract

Pregnancy‐associated cervical polyps have been linked to an increased risk of spontaneous late miscarriage and preterm delivery. In particular, bleeding cervical polyps, often suspected to be decidual in origin, may represent a high‐risk condition requiring clinical intervention. We report a case series of three pregnant women with bleeding cervical polyps in whom decidual polyps were suspected based on clinical and ultrasonographic features. In all cases, targeted cauterization of the polyp surface was performed primarily to achieve hemostasis under adjunctive administration of oral synbiotic therapy. This approach resulted in immediate hemostasis and was followed by gradual regression and complete macroscopic disappearance of the polyps. All three pregnancies resulted in term deliveries. Targeted cauterization may represent a minimally invasive management option for bleeding cervical polyps during pregnancy, although further studies are needed.

## Key Clinical Message

Targeted cauterization may provide effective hemostasis in selected cases of bleeding cervical polyps during pregnancy and may be followed by regression of the lesion. This minimally invasive approach may be associated with favorable pregnancy outcomes, although further evidence is required.

## 1 Introduction

Pregnancy‐associated cervical polyps have been reported to increase the risk of spontaneous late miscarriage and preterm delivery (sLMC/PTD), regardless of whether the polyps are removed [1, 2, 3, 4, 5]. Conventional polypectomy by grasping and twisting (torsional removal) using Kelly or Pean forceps has been widely performed; however, this procedure does not appear to reduce these risks and may even exacerbate them [1, 2, 3].

Ascending infection from the vaginal microbiota has been suggested as one of the possible mechanisms underlying these adverse outcomes. Therefore, in our institution, pregnant women with cervical polyps are routinely managed with adjunctive therapy aimed at supporting vaginal *Lactobacillus* dominance and maintaining vaginal homeostasis [6, 7, 8]. This regimen includes oral lactoferrin (300 mg/day) and probiotics containing *Clostridium butyricum* , *Enterococcus faecium* , and *Bacillus subtilis* , administered using a commercially available formulation approved in Japan. For simplicity, this combined adjunctive regimen is hereafter referred to as synbiotic therapy.

However, certain clinical features have been associated with an increased risk of sLMC/PTD, including suspected decidual polyps, bleeding from the polyp, and large polyp size [1, 2]. These situations require careful and individualized management. We previously reported that ligation‐induced necrosis may be an effective minimally invasive option for large cervical polyps during pregnancy [9]. In contrast, optimal management strategies for bleeding cervical polyps remain unclear, particularly when immediate hemostasis is required.

In such cases, we explored an additional minimally invasive approach targeting the polyp surface. Cauterization was performed using a loop electrosurgical excision procedure (LEEP) generator in coagulation mode with a ball electrode at a power setting of 40–50 W. No local anesthesia was used, and no cervical dilation or manipulation of the cervical canal was performed. Superficial coagulation was applied to the bleeding surface of the polyp, with several applications lasting a few seconds each to achieve hemostasis. No laser or chemical cauterization (e.g., silver nitrate) was used. These interventions were undertaken as part of individualized clinical management in routine practice rather than within a formal research protocol.

The aim of this case series was to describe the clinical course and outcomes of bleeding cervical polyps during pregnancy managed with targeted cauterization for hemostasis and subsequent polyp regression. Because the procedure can be completed within seconds and performed in an outpatient setting, this technique may represent a simple and minimally invasive management option.

## 2 Case Series

### 2.1 Case 1

**FIGURE 1:**
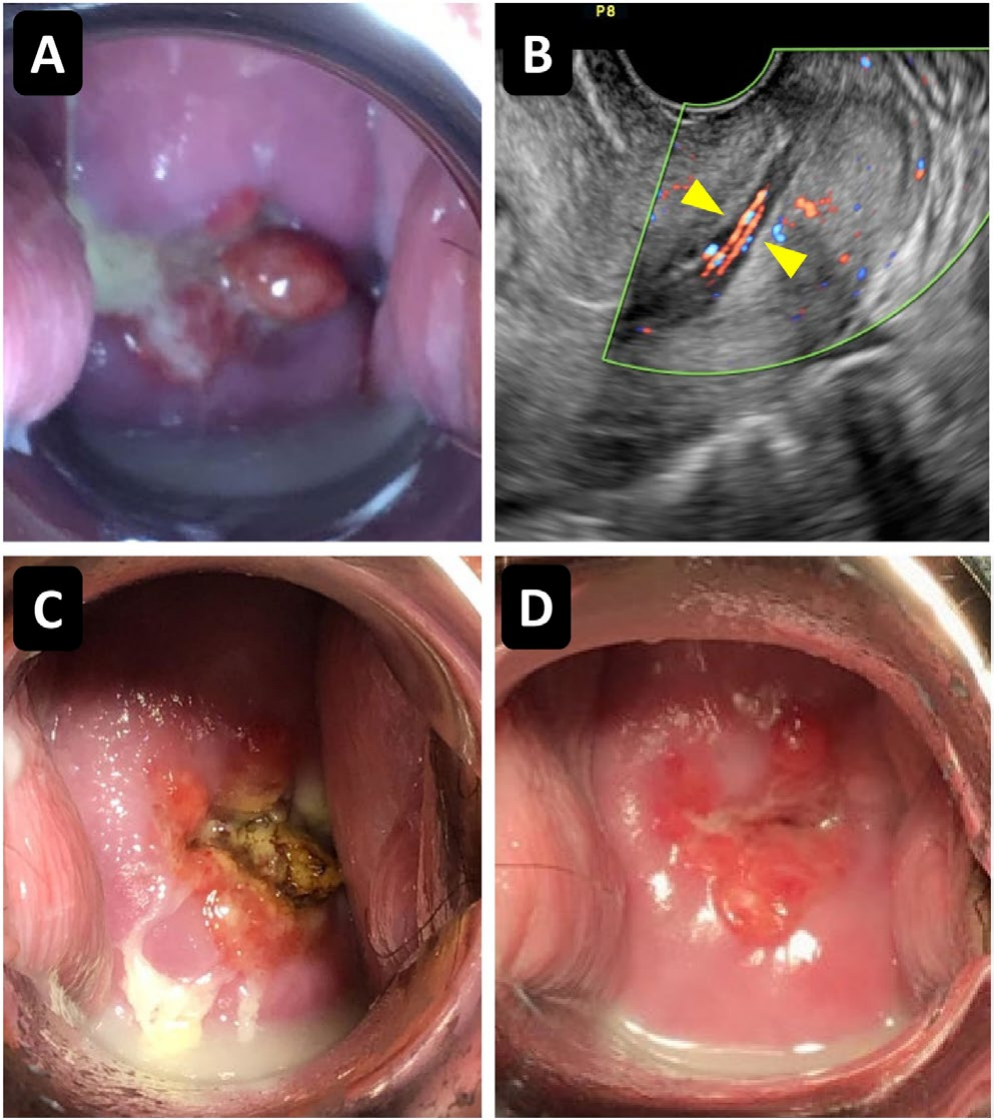
Case 1. (A) At 10 weeks and 4 days of gestation, a cervical polyp protruding from the external cervical os, measuring 10 mm in diameter, was observed. (B) Color Doppler ultrasonography showed blood flow within the cervical canal continuous with the stalk of the cervical polyp (yellow arrows), supporting the diagnosis of a decidual cervical polyp. (C) At 11 weeks and 3 days of gestation, cauterization was performed, consisting of five applications of 2–3 s each, resulting in hemostasis. After cauterization, a superficial eschar formed on the polyp surface, without thermal extension into the cervical canal. (D) At 19 weeks and 5 days of gestation, the cervical polyp was no longer visible on macroscopic examination.

A pregnant woman in her 30s (gravida 3, para 1) with no history of sLMC/PTD presented with slight vaginal bleeding at 10 weeks and 4 days of gestation. Transvaginal examination revealed a cervical polyp measuring 10 mm in diameter (Figure 1A). Color Doppler ultrasonography demonstrated vascularity within the stalk (Figure 1B), suggesting a decidual cervical polyp. In accordance with our institutional management strategy, adjunctive synbiotic therapy was initiated.

At 11 weeks and 3 days of gestation, targeted cauterization of the bleeding polyp surface was performed primarily to achieve hemostasis, consisting of five applications of 2–3 s each, resulting in immediate bleeding control (Figure 1C). At 13 weeks and 4 days of gestation, the polyp had regressed to approximately 5 mm in diameter. Doppler ultrasonography no longer detected blood flow within the stalk, and bleeding did not recur. By 19 weeks and 5 days of gestation, the cervical polyp was no longer macroscopically visible (Figure 1D).

At 21 weeks and 4 days of gestation, the cervical length measured 46 mm, and the pregnancy progressed without signs of sLMC/PTD. Synbiotic therapy was continued until 36 weeks and 6 days of gestation. The patient delivered a healthy female infant weighing 3516 g at 39 weeks and 3 days of gestation via vaginal delivery. The maternal and neonatal postpartum courses were uneventful.

### 2.2 Case 2

A pregnant woman in her 30s (gravida 3, para 1) had a history of cervical polypectomy by torsional removal at 8 weeks of gestation in a previous pregnancy, followed by cervical insufficiency and stillbirth at 21 weeks. Placental pathology had revealed histological chorioamnionitis (stage 3).

**FIGURE 2:**
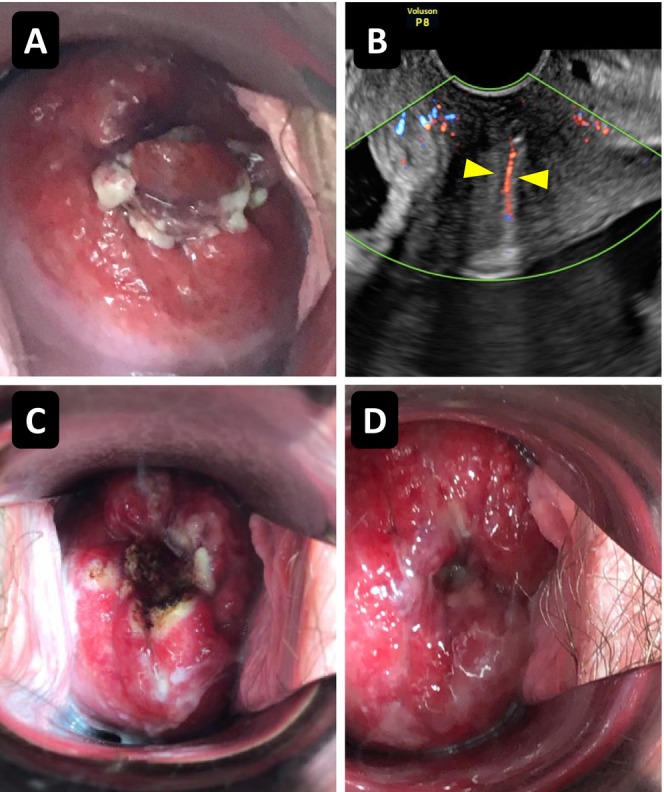
Case 2. (A) At 8 weeks and 6 days of gestation, three cervical polyps measuring 12, 8, and 5 mm in diameter were observed, accompanied by bleeding from the polyps. (B) Color Doppler ultrasonography showed blood flow within the stalk of the cervical polyp located in the cervical canal (yellow arrows). (C) At 12 weeks and 6 days of gestation, cauterization was performed, consisting of five applications of 2–3 s each, resulting in successful hemostasis. After cauterization, a superficial eschar formed on the polyp surface, without thermal extension into the cervical canal. (D) At 14 weeks and 6 days of gestation, the cervical polyps were no longer visible on macroscopic examination.

In the current pregnancy, at 8 weeks and 6 days of gestation, three cervical polyps measuring 12, 8, and 5 mm were identified (Figure 2A). Color Doppler ultrasonography demonstrated blood flow within the stalks (Figure 2B), strongly suggesting decidual cervical polyps. Adjunctive oral synbiotic therapy was initiated.

At 10 weeks and 6 days of gestation, mild vaginal bleeding occurred. At 12 weeks and 6 days, targeted cauterization of the bleeding polyp surface was performed to achieve hemostasis, consisting of five applications of 2–3 s each, resulting in complete bleeding control (Figure 2C). Because slight bleeding persisted one week later, additional cauterization was performed at 13 weeks and 6 days, consisting of two applications of 2–3 s each. At 14 weeks and 6 days of gestation, the cervical polyps were no longer macroscopically visible (Figure 2D).

At 20 weeks and 6 days, the cervical length measured 34 mm. Synbiotic therapy was discontinued at 36 weeks and 6 days. The patient delivered a female infant weighing 2856 g at 38 weeks and 0 days of gestation via vaginal delivery. Both the mother and infant had uncomplicated postpartum courses.

### 2.3 Case 3

**FIGURE 3:**
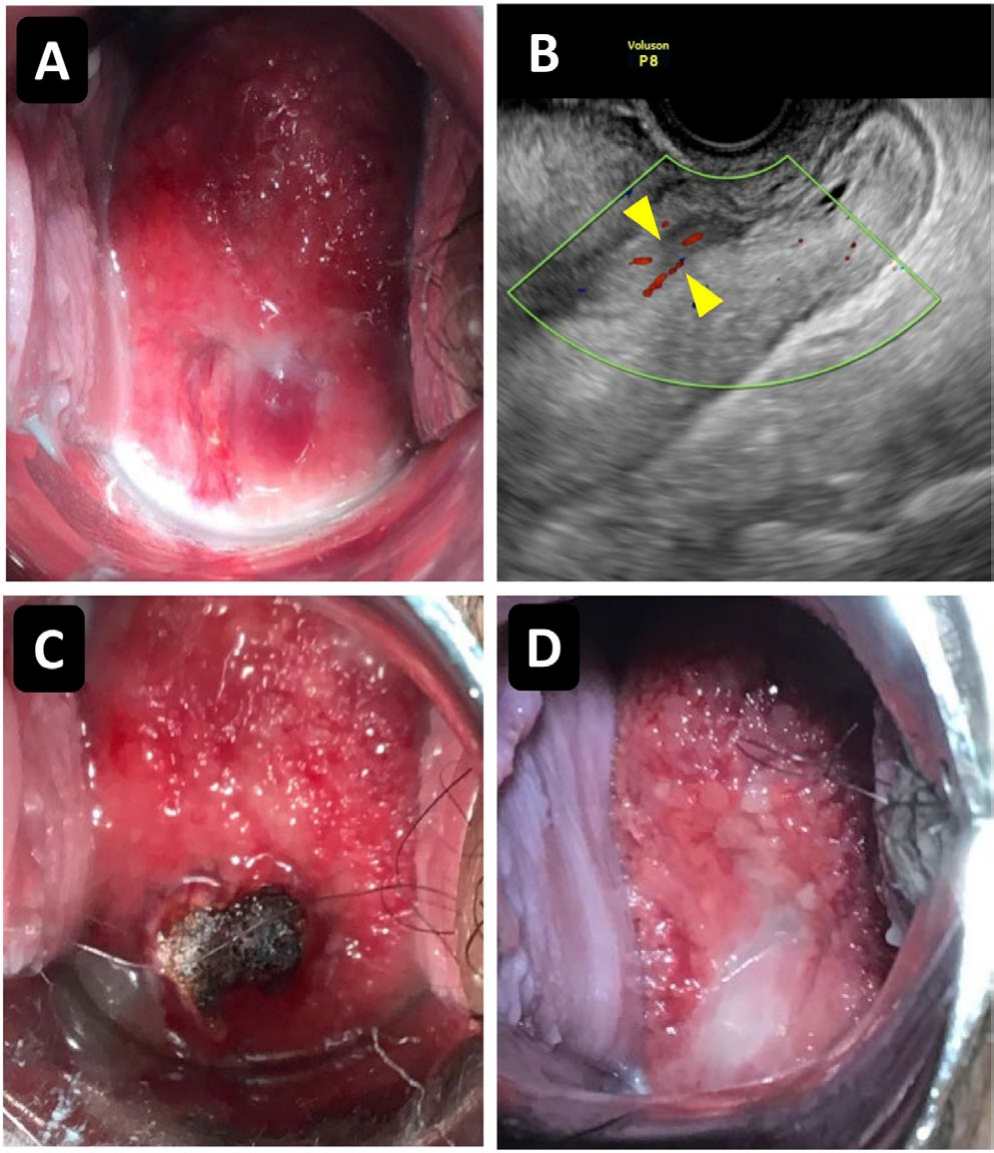
Case 3. (A) At 11 weeks and 3 days of gestation, two cervical polyps measuring 8 and 6 mm in diameter were observed, accompanied by bleeding from the polyps. (B) Color Doppler ultrasonography showed blood flow within the stalks of the cervical polyps located in the cervical canal (yellow arrows). (C) At 12 weeks and 4 days of gestation, cauterization was performed, consisting of three applications of 2–3 s each, resulting in successful hemostasis. After cauterization, a superficial eschar formed on the polyp surface, without thermal extension into the cervical canal. (D) At 13 weeks and 4 days of gestation, the cervical polyps were no longer visible on macroscopic examination.

A pregnant woman in her 30s (gravida 3, para 1) with a history of cesarean delivery for breech presentation had two uterine fibroids measuring approximately 2 cm in diameter. At 11 weeks and 3 days of gestation, two cervical polyps measuring 8 and 6 mm, with bleeding from the polyp surfaces, were identified (Figure 3A). Color Doppler ultrasonography showed blood flow within the stalks (Figure 3B), suggesting decidual cervical polyps. Adjunctive oral synbiotic therapy was initiated.

At 12 weeks and 4 days of gestation, targeted cauterization of the bleeding polyp surface was performed primarily to achieve hemostasis, consisting of three applications of 2–3 s each, resulting in immediate bleeding control (Figure 3C). At 13 weeks and 4 days, the cervical polyps were no longer macroscopically visible (Figure 3D).

At 21 weeks and 3 days of gestation, the cervical length measured 44 mm. Synbiotic therapy was discontinued at 36 weeks and 6 days. At 38 weeks and 2 days of gestation, the patient underwent cesarean delivery because of the previous cesarean section and delivered a female infant weighing 3202 g, with Apgar scores of 8 and 9 at 1 and 5 min, respectively. The postpartum course was uneventful.

## 3 Discussion

In our previous study summarizing the outcomes of pregnant women with cervical polyps managed by conventional torsional removal, cervical polyps accompanied by bleeding during pregnancy were associated with a high risk of sLMC/PTD [2]. These findings highlight the need for careful and individualized management, particularly in cases with bleeding.

While modulation of the vaginal microbiota may play an important role in preventing ascending infection and/or inflammation, this strategy alone may be insufficient in selected high‐risk cases, such as cervical polyps suspected to be decidual in origin or polyps with bleeding originating from the polyp itself. In such cases, an additional intervention targeting the polyp itself, while minimizing cervical trauma, may be clinically justified. In this context, we focused on targeted cauterization as a minimally invasive therapeutic option for selected cases of bleeding cervical polyps during pregnancy.

In the three cases presented herein, targeted cauterization performed primarily for hemostasis resulted in prompt bleeding control and gradual regression of the polyps, ultimately leading to macroscopic disappearance. No major procedure‐related complications were observed, such as significant pain, burning sensation, infection, cervical shortening, or persistent bleeding. In one case, mild recurrent bleeding occurred one week after the initial cauterization and was successfully controlled with an additional superficial cauterization. A plausible mechanism is that cauterization reduced vascular supply to the polyp tissue, leading to progressive attenuation of blood flow within the feeding vessels of the stalk and subsequent regression of the lesion.

Although histological confirmation was not available, the diagnosis of decidual cervical polyps was considered presumptive and based on clinical features. These included early gestational presentation, an irregular polyp surface that appeared slightly friable and bled easily on contact, marked vascularity within the stalk, and spontaneous regression during pregnancy. Nevertheless, other conditions should be considered in the differential diagnosis, including endocervical polyp, cervical ectropion‐related lesions, other vascular cervical lesions, cervical malignancy, and cervical leiomyoma.

The potential impact of oral synbiotic therapy on pregnancy outcomes cannot be determined in this case series. Although vaginal *Lactobacillus* dominance has been associated with a reduced risk of sLMC/PTD, the evidence remains limited [10, 11, 12]. It is possible that a *Lactobacillus*‐dominant vaginal environment may help suppress ascending infection. Both targeted cauterization for local hemostasis and regression of the polyp, and maintenance of a *Lactobacillus*‐dominant vaginal microbiota may have contributed to the favorable outcomes observed in these cases, and their relative contributions cannot be determined.

In all three cases, cervical length was monitored every 1–2 weeks after the procedure. No post‐procedure uterine contractions or additional obstetric interventions were observed. Routine systemic inflammatory markers such as white blood cell count or C‐reactive protein were not measured. Vaginal microbiological assessment and cervical inflammatory evaluation were performed as part of routine care, but these findings were not systematically analyzed in this case series.

Management of cervical polyps during pregnancy remains controversial. Previous systematic reviews have reported that both expectant management and polypectomy may be associated with an increased risk of adverse obstetric outcomes, including sLMC/PTD, regardless of whether the polyp is removed [13, 14]. Some authors have suggested that, if polypectomy is performed, it may be safer after 12 weeks of gestation [13, 14]. In contrast, the present approach does not involve removal of the polyp itself. Instead, targeted cauterization is performed primarily for hemostasis. In our cases, this minimally invasive procedure successfully controlled bleeding, and the polyps subsequently regressed spontaneously during pregnancy. In clinical practice, targeted superficial cauterization may be considered in selected cases of bleeding cervical polyps during early pregnancy, particularly when Doppler ultrasonography demonstrates a vascular stalk within the cervical canal and the lesion is highly prone to bleeding. This approach may provide effective hemostasis while minimizing cervical manipulation. However, further studies are needed to clarify the relative benefits of this strategy compared with torsional polypectomy or expectant management.

Although electrosurgical techniques have been widely used in obstetric and fetal therapy [15, 16], the present approach was limited to superficial cauterization of cervical polyps outside the uterine cavity. The procedure was brief and could be performed in an outpatient setting. Repeated cauterization may also be considered according to the clinical course, making this approach practical for both hemostasis and gradual polyp reduction.

This case series describes clinical management performed as part of routine medical care. According to the policies of our institutional ethics committee, formal institutional review board approval was not required for this type of case report. Written informed consent was obtained from all patients for treatment and for publication of the clinical details and images.

This study has several limitations. First, the number of cases was small, and all cases were managed at a single center, which limits the generalizability of the findings. Second, histological confirmation of the polyps was not available, and the diagnosis of decidual polyps was based on clinical features. Third, because probiotics and lactoferrin were administered concurrently, their potential contribution to the observed outcomes cannot be completely excluded.

Nevertheless, targeted cauterization of the surface of bleeding cervical polyps during pregnancy may represent a feasible and minimally invasive management option in carefully selected cases. In this series, cauterization achieved effective hemostasis and was followed by polyp regression, with favorable pregnancy outcomes observed in all three patients. Further studies are warranted to confirm the safety and clinical efficacy of this approach.

## Author Contributions

**Satoshi Yoneda:** conceptualization, data curation, visualization, writing – original draft, writing – review and editing. **Noriko Yoneda:** writing – review and editing. **Tatsuhiro Tsuda:** writing – original draft. **Masami Ito:** writing – review and editing. **Sayaka Tsuda:** writing – review and editing. **Kyoko Takemura:** conceptualization, writing – review and editing.

## Funding

This study was supported by a Grant‐in‐Aid for Scientific Research from the Japan Society for the Promotion of Science (JSPS KAKENHI Grant 25K12697).

## Ethics Statement

This case series describes clinical management performed as part of routine medical care. According to the policies of our institutional ethics committee, formal institutional review board approval was not required for this type of case report.

## Consent

Written informed consent was obtained from all patients for treatment and for publication of this case series and the accompanying images.

## Conflicts of Interest

The authors declare no conflicts of interest.

## Data Availability

The data supporting the findings of this study are available from the corresponding author upon reasonable request.
